# The Utility of [18]F-Fluorocholine Positron Emission Computed Tomography and [18]F-Fluorodeoxyglucose Positron Emission Tomography–Computed Tomography in Evaluating Breast Cancer Phenotypes: A Pilot Study

**DOI:** 10.5152/eurasianjmed.2024.23047

**Published:** 2024-06-01

**Authors:** Mohd Hazeman Zakaria, Shazreen Shaharudin, Fathinul Fikri Ahmad Saad

**Affiliations:** 1 Centre for Diagnostic Nuclear Imaging, Universiti Putra Malaysia Faculty of Medicine and Health Science, Selangor, Malaysia; 2 Universiti Pertahanan Nasional Malaysia, Kuala Lumpur, Malaysia

**Keywords:** [18]F-fluorocholine, HER 2 negative, [18]F-FDG PET-CT, phenotypes, globals health status

## Abstract

**Background:**

The utility of the [18]F fluorodeoxyglucose positron emission tomography–computed tomography ([18]F FDG PET-CT) marker for breast cancer is well established. Given its limitations in localizing FDG-negative malignant tumors, the expression of [18]F-fluorocholine ([18]-FCH) may potentially be helpful to improve the overall accuracy in evaluating breast cancer. This study determined the potential of [18]-FCH PET CT as a potential marker in assessing breast cancer phenotypes.

**Methods:**

We recruited consecutive patients with biopsy-proven breast carcinoma who underwent [18]F-FCH PET-CT following the [18]F-FDG PET-CT imaging. The subjects were dichotomized into human epidermal growth factor receptor 2 (HER2)-negative and HER2-positive genotypes. The maximum standardized uptake value (SUV_max_; g/dL) was used to predict the two groups of variables. Global health status (GHS) score based on the EORTC quality of life questionnaire (QLQ) was used to evaluate the outcome of the cohort subjects at 6, 12, and 24 months.

**Results:**

There were 21 females with a mean age of 54.48 ± 12.17 years. Eighteen patients had invasive ductal carcinoma (18/21;85.8%) on histology, with 11 (52.4%) were HER2-negative genotype. There was higher sensitivity and specificity of [18]-FCH-PET/CT in breast lesions at 40% and 68.8% compared to [18]FDG-PET/CT with 33.3% and 66.7%, respectively. There were significant differences between [18]F-FCH SUV_max_ (g/dL) of the HER-negative as compared to the HER2- positive group (1.99 g/dL vs. 0.2 g/dL; *P* < .05). High SUV_max_ (g/dL) of [18]F-FCH had predicted the HER-negative genotype at the cutoff value of 0.75 (*P* < .05). High [18]F-FCH showed significantly poor scoring of GHS parameters compared to low FCH at 6 months (mean SUV_max_ 8.06 vs. 5.40 respectively; *P* < .05).

**Conclusion:**

[18]F-FCH PET-CT is a potential marker in localizing and predicting aggressive breast carcinoma phenotypes.

**Main Points**

- [18]F-fluorocholine (FCH) shows promise as a potential specific marker for detecting overexpression of the choline receptor metabolism.
- [18]F-FCH is potentially a complementary marker to [18]F-fluorodeoxyglucose (FDG) in localizing and predicting potentially poor FDG avidity of the subcentimeter breast tumor lesions.
- A higher [18]F-FCH maximum standardized uptake value (SUVmax) predicted a poorer quality of life when the SUV_max_ value recidivates 0.75.

## Introduction

The incidence of breast cancer is increasing, affecting more than 1 million women worldwide. In Malaysia, breast cancer incidence accounted for 34.1% of all cancers among females in Malaysia.^1^ Recent research showed that half of breast cancers develop in women who have no identifiable breast cancer risk factor other than gender (female) and age (over 40 years).^2^ Human epidermal growth factor receptor 2 (HER2)-negative phenotypes, regardless of estrogen or progesterone status, have been reported as a marker for aggressive breast cancer. Few studies have evaluated the association of HER2-negative breast cancer phenotypes with cell membrane metabolism via choline kinase cellular reprogramming.^3-4^ Gene expression based on the intrinsic classification of breast cancer can be categorized into 4 subtypes: luminal A, luminal B, typical breast-like HER2, and a basal-like subtype.^3^ Changes in the expression of various genes have resulted in significantly different clinical outcomes due to alterations in the cellular pathways and other mutational profiles that reflect the tumor microenvironment.

Overexpression of the HER2 histologic marker indicates a lower overall survival and shorter disease-free interval than patients without such expression. Moreover, overexpression of estrogen, progesterone, and HER2 leads to aggressive breast cancer. HER2 (or HER2/neu) is also known as an ERBB2 or receptor tyrosine-protein kinase erB-2 protein that is encoded by the ERBB2 gene in humans. Overexpression of ERBB2 occurred in approximately 15%-30% of breast cancers and was strongly associated with recurrence, the aggressiveness of tumors, overall survival, and a poor prognosis.^4^ The association between choline metabolism and this histologic marker is not entirely understood, even though there are reports on the transfection of human breast tissue cells with the HER2 gene, which has been known to cause an increase in phosphocholine.^5^

The [18]F-fluorodeoxyglucose (FDG) marker has been widely used to identify malignant cellular breast carcinoma as high FDG accumulation in a malignant cell denotes an aggressive tumor.^5^ The use of FDG positron emission tomography–computed tomography (PET-CT) in evaluating breast cancer is well established; however, it has limitations. [18]F-FDG has poor sensitivity in localizing the malignant types of breast cancer, particularly the lobular type and small-volume lesions.^5-6^

Furthermore, it is not specific, as inflammation and inflammatory lesions commonly produce false-positive results. Previously, [18]F-fluorocholine (FCH) PET-CT has been used for oncological imaging in prostate and brain cancer.^7^ The expression of choline tracer metabolism is an essential mechanism for the staging and treatment of breast cancer due to the discovery.^6-8^ The transformation of human breast cancer is associated with a higher level of creatine kinase that is responsible for the catalysis of phosphorylated choline to phosphocholine.^9-10^ Nevertheless, there is still inadequate substantial or decisive data to suggest the feasibility and efficacy of choline in becoming a surrogate marker for signaling breast cancer cell metabolism in breast cancer diagnosis and treatment. Thus, this study will document an essential discovery of the potential utility of [18]F-FCH in aggressive breast cancer genotypes.

## Material and Methods

**Figure 1.**
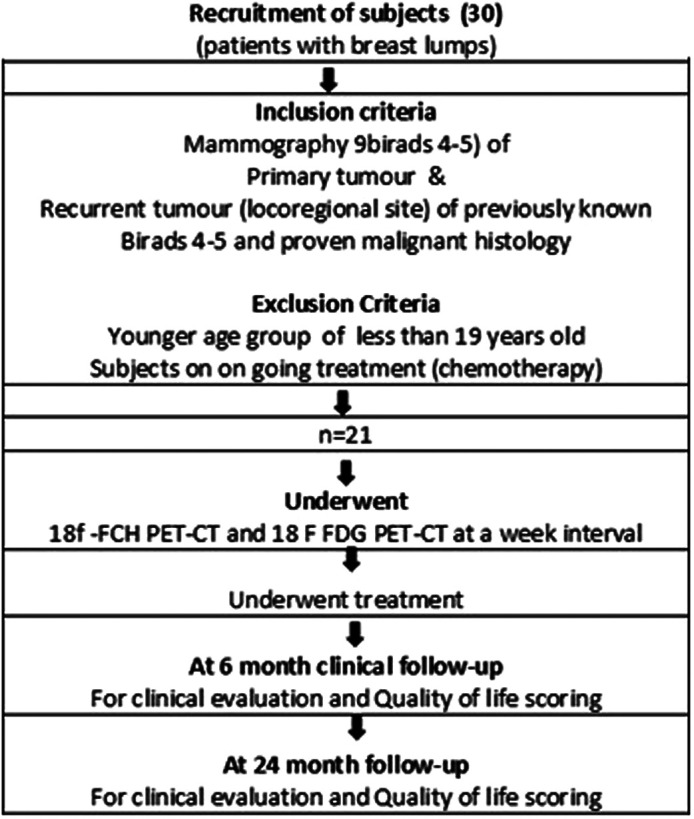
Patients’ workflow in this study. PET-CT, positron emission tomography–computed tomography.

Thirty consecutive subjects underwent a mammogram for a suspicious breast lump. Informed consent was obtained from patients who participated in this study. All subjects with primary breast lesions and a previously proven malignant tumor (Birads 4-5 lesions) who presented with a loco-regional lump on the same side of the breast on clinical follow-up were included. This study was approved by the Putra malaysia university’s ethics committee (Approval date: 21st Nov 2014 approval no: JKEUPM/F2). Informed consent was obtained from the patients who agreed to take part in the study. Based on inclusion and exclusion criteria (Figure 1), only 21 subjects were included following confirmation of malignant histology by the diagnostic [18]F FDG and [18]F FCH and the immunohistological analysis. The subjects underwent [18]F-fluorocholine PET-CT, followed by [18]F FDG PET-CT after an average of a 1-week interval. The cohort of subjects was followed up at 6 months, 12 months, and 24 months following treatment (Figure 1).

## [18]F-Fluorodeoxyglucose Positron Emission Tomography–Computed Tomography

Patients were required to fast for 6-8 hours before examination. They were only allowed to drink water. Patients with diabetes mellitus or hypertension were instructed to take their medication on the morning of the examination. The fasting sugar level was less than 10 mmol/L (European Association of Nuclear Medicine for PET). According to the European protocol, the administered dose of [18]F-FDG ranges between 296 and 370 MBq (8-10 mCi) through intravenous access through the arm veins. After injection, the patient remained supine and rested for approximately 50 minutes before being subjected to PET-CT.

### [18]F-Fluorocholine Positron Emission Tomography–Computed Tomography

After successful [18]F-FDG PET/CT, the patients were scheduled for [18]F-FCH PET/CT studies 1 week later. 3-5 mci/kgbody weight (BW) or 111-185 Mbq/kg of [18]F-FCH was given through the IV line. The patients did not need fasting for [18]F-FCH PET/CT examination. The patient rested for 10 minutes to allow an excellent choline’s blood before being subjected to PET/CT scanning. The PET-CT scanning was performed with the patient in a supine position, adopting a shallow breathing technique. This study’s PET/CT examination was performed using a dedicated integrated PET/CT system (Siemens Biograph-64, Erlangan, Germany) consisting of 2 structural and functional image information detection components. The PET emission scan was produced using 3-dimensional imaging. The imaging of the PET system gives an efficient ejection of random events, high light output and count rates, and fast coincidence timing.

#### Image Interpretation

For image fusion and generation of CT transmission maps, data were re-sized from a 512 × 512 matrix to a 128 × 128 matrix. The ordered subsets’ expected maximization was used to reconstruct PET images with segmented measured attenuation correction using CT data with 28 subsets and two iterations. A 5 mm FWHM (Full width at half maximum) Gaussian filter was used for post-reconstruction smoothing of images.

#### Qualitative Positron Emission Tomography–Computed Tomography Image Interpretation

### Grade of Fluorocholine Uptake/Kerning

**Figure 2.**
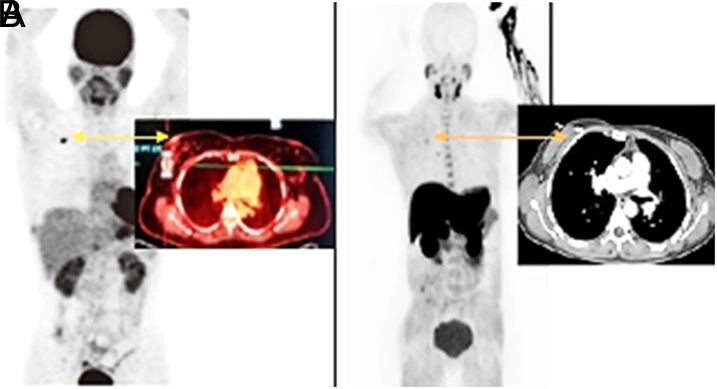
Coronal (A) Multiple Image Projection (MIP) [18]F-fluorodeoxyglucose (FDG) positron emission tomography–computed tomography (PET-CT) image showing FDG-avid right breast lesion with SUV_max_ -5.5 g/dL compared to the mediastinal blood pool—positive lesion—coronal MIP (B). MIP PET and axial CT showed a low intense fluorocholine (FCH) uptake of the right breast lesion compared to the mediastinal blood pool—positive lesion. The added value of 18F FCH is that it serves to complement changes of altered glucose metabolism on the [18]F-FDG by exhibiting changes of altered lipid metabolism in a malignant cell, hence strengthening evidence of an actual mitotic lesion. SUV, standardized uptake value.

To interpret the radiotracer of [18]F-FCH uptake within the primary lesion, a 3-point visual scoring system was used (Figure 2). This system is compared with the mediastinal blood pool. A low grade direct lesion uptake is less than the mediastinal blood pool. A moderate primary lesion uptake is equal to mediastinal blood pool activity. A high grade immediate lesion uptake is more elevated than mediastinal blood pool activity.

### Semiquantitative Measurement

The dedicated PET/CT scanner was developed to measure the in vivo radioactivity concentration (kBq/mL) within tissue uptake of FDG and FCH. The uptake varies depending on injected amount, duration, and weight. A standardized uptake value (SUV) was used to measure FCH uptake. The range value of SUV_max_ (g/dL) for [18]F-FCH PET/CT is relatively low compared to FDG. In the study by Knee et al,^11^ the value of SUV for [18]F-FCH PET/CT is inferior to FDG, which probably concerns its metabolism and bioavailability.

### Immuno- Histopathological Examination—Gold Standard

#### Immunohistology/Breast Cancer Phenotype and Classification of Tumor Immunohistochemical Subtypes

Breast cancer has heterogeneous molecular phenotypes (classified as in situ or invasive carcinomas) that determine the patient’’s staging, prognosis, and outcome. In this study, we dichotomized tumor aggressiveness into HER2-negative (HER2 −ve) (aggressive) and HER2-positive (HER2 +ve) (nonaggressive). The HER2 −ve phenotype is a reliable prognostic biomarker for breast cancer receptor characteristics.^11-14^ addition, the HER2 −ve marker, regardless of estrogen (ER) or progesterone (PR) status, has been reported as a marker for aggressive breast cancer phenotypes.^15-16^ Based on the imaging criteria, staging was also dichotomized into low stage (stage I/II) and high stage (stage III/IV).

### Quality of Life Parameter

**Figure 3.**
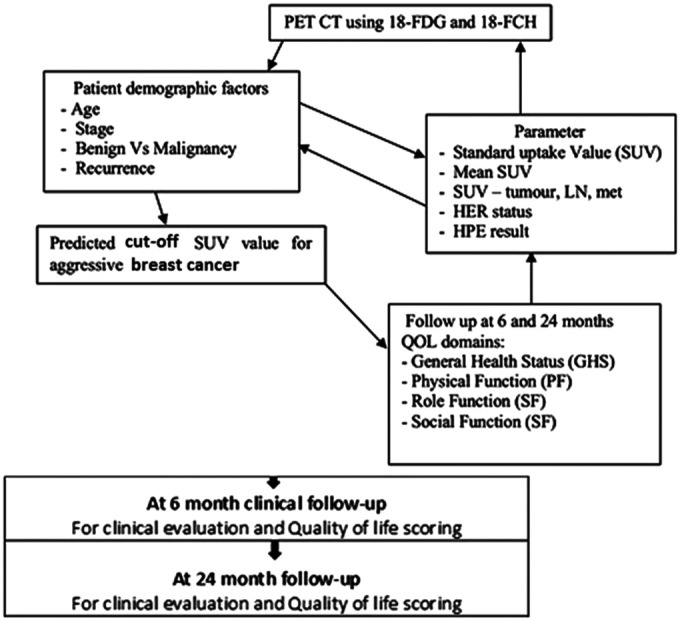
Conceptual framework showing an association between patient demographic factors, parameters, predicted standardized uptake value value and quality of life domains. FCH, fluorocholine; FDG, fluorodeoxyglucose; PET-CT, positron emission tomography–computed tomography; SUV, standardized uptake value.

Patient-experience data, such as those from quality of life (QOL) instruments, can be valuable in risk–benefit assessment. It consists of essential elements of the general well-being of individuals, families, communities, and society (Figure 3). When evaluating changes in breast cancer burden scores among the 21 subjects throughout therapy, a 5-point threshold (good--poor) has been used to interpret the meaningfulness of within-patient score changes. In this regard, the cohort of 21 subjects was followed up 6 months after the initial treatment to assess the QOL parameter as part of the clinical progress. It was followed at 12 months and 24 months. The parameters evaluated the range of negative and positive outcomes as predicted from test variables, i.e., biochemical markers, imaging markers, and the type of treatments received based on the subjects’ responses about personal satisfaction.^17^

### Treatment Regimens

Six subjects were dropped out during the follow-up. We dichotomized the remaining 15 subjects into those with surgery and radiation therapy, chemotherapy, and targeted therapy. Surgery as initial treatment is appropriate for those with smaller, node-negative tumors. In this cohort, surgery alone was offered for patients with localized lesions, and it is crossfire with radiation therapy for the adjoining lymph node metastasis. Patients with HER2-negative non-metastatic breast cancer generally warrant adjuvant treatment with chemotherapy and trastuzumab. human epidermal growth factor receptor 2-negative metastatic patients all benefited from chemotherapy. In this cohort, 11 subjects had there undergone targeted treatment and chemotherapy only, and only four subjects had surgery alone and radiation therapy.

### Statistical Analysis

Statistical analyses were performed using IBM SPSS® Statistics, version 26 (IBM SPSS Corp.; Armonk, NY, USA). Descriptive statistics were utilized for selected variables. Socio-demographic analysis of parameters such as age group, mammogram BIRADS, and biopsy results was done using univariate descriptive analysis. All numerical data were described using means and standard deviation depending on the distribution of the respective variables. Two groups were compared using an independent *t*-test. Qualitative data were analyzed using the chi-square test and Kendall’s Tau. All statistical tests were considered significant with *P*-values less than .05.

## Results

The 21 subjects included had a mean age of 54.48 ± 12.17 years. All patients had invasive ductal carcinoma: 11 subjects with single ER/PG – HER2 −ve and 10 with single ER/PG—HER2 +ve (Table 1).

Table 1 shows the accuracy of [18]F-FCH PET-CT versus [18]F-FDG PET-CT.

**Figure 4.**
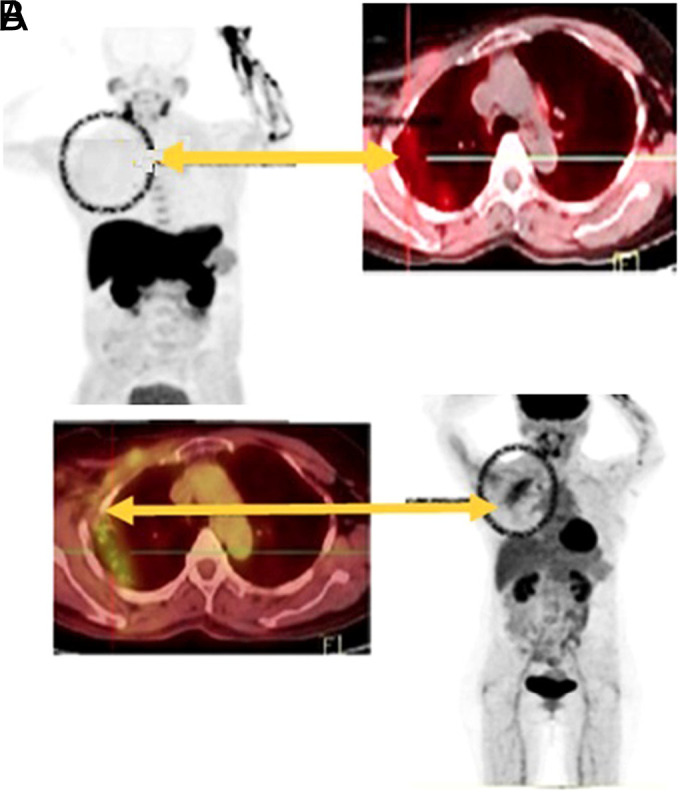
A 60-year-old female with human epidermal growth factor receptor 2 +ve right breast tumor (T3N0M1) post right mastectomy and radiotherapy. Coronal (A) MIP positron emission tomography (PET) (A): [18]F-fluorocholine (FCH) revealed no evidence of altered phospholipid metabolism denoting radiation pneumonitis as shown on the [18]-F-fluorodeoxyglucose (FDG) image axial (B) fused PET-computed tomography. The added value of the [18]F-FCH is that it dismisses false-positive changes of a metastatic tumor as noted on the correlated FDG-avid radiation pneumonitis.

The sensitivity and specificity of [18]F-FCH PET-CT and [18]F-FDG PET-CT in determining breast cancer aggressiveness based on histopathology were compared. There was higher sensitivity and specificity of [18]-FCH PET-CT in breast lesions, at 40% and 68.8%, compared to [18]F-FDG PET-CT at 33.3% and 66.7%, respectively (Figure 4A-B). Nevertheless, higher sensitivity and specificity for lymph node lesions and distant metastasis were noted for [18]F-FDG PET-CT compared to [18]F-FCH PET-CT (Table 2).

Table 2 shows the sensitivity and specificity of [18]F-FCH PET-CT compared to [18]F-FDG PET-CT in breast, lymph node, and metastasis.

The association of [18]F-FCH PET-CT and [18]F-FDG PET-CT with breast lesions.

There was a significant difference in the mean SUV_max_ of metastatic lesions in [18]F-FCH PET-CT compared to [18]F-FDG PET-CT (2.27 ± 3.19 g/dL vs. 1.74 ± 2.32 g/dL, *P *= .004, respectively). There was no significant difference between [18]F-FDG and [18]F-FCH among the primary tumor lesions of both the breast and lymph node lesions (Table 3).

Table 3shows the [18]F-FCH and [18]F-FDG mean and standard deviation for patients with breast lesions, mixed-side lymph node lesions, and metastasis.

The association of [18]F-FCH PET-CT and [18]F-FDG PET-CT with HER 2 phenotypes

**Figure 5.**
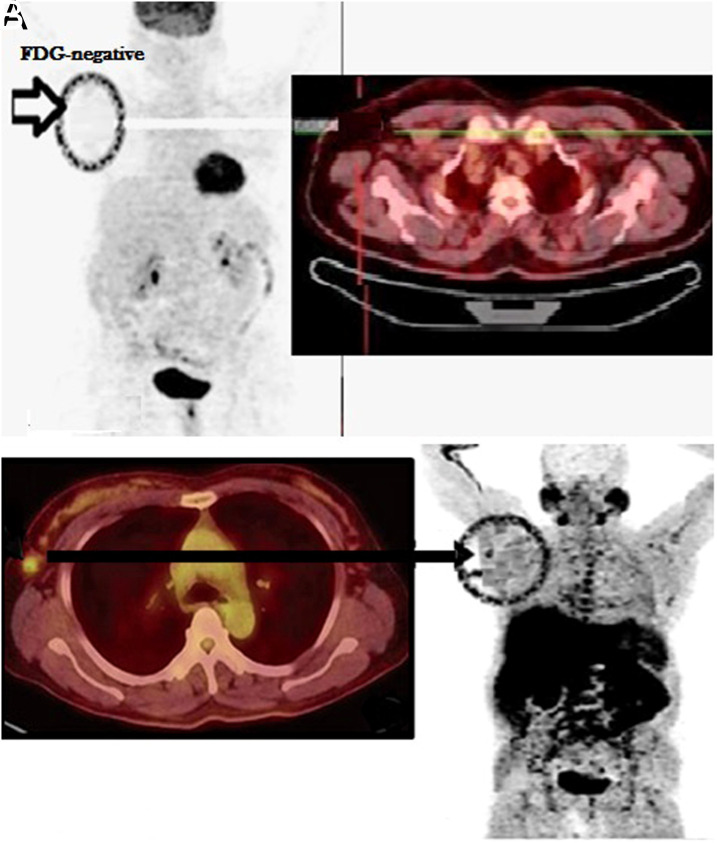
A 51-year-old lady with single ER/PG human epidermal growth factor receptor 2 negative. Coronal (A) MIP positron emission tomography (PET). Fluorodeoxyglucose-negative right axillary lymph node deposit. Axial (B) fused PET-computed tomography (CT): Fluorocholine (FCH)-positive right axillary lymph node. The added value of 18F FCH-avid right axillary lymph node is its higher sensitivity than 18[F]-FDG in a small nodal metastasis deposit.

There was a significant difference in the overall mean [18]F-FCH SUV_max_ (g/dL) of the Single ER/PG HER2 −ve and the ER/PG HER2 +ve patients (1.99 ± 1.52 g/dL vs. 0.2 ± 0.22 g/dL; *P* < .05) (Table 4). The ER/PG HER2 −ve mean of the FCH-imaged lesions showed a higher value than [18]F-FDG. Most of the subcentimeter lymph node lesions among the [18]F-FCH phenotype exhibited higher intrinsic [18]F-FCH uptake than [18]F-FDG uptake (Figure 5A and B).

Table 4 shows the prediction of the overall mean SUV_max_ of [18]FCH (g/dL) and [18]F-FDG on the single ER/PG HER2 status.

Table 5 shows the prediction of the overall SUV mean of Single ER/PG HER on [18]F-FDG and [18]F-FCH.

There was also a significant difference in the mean [18]F-FDG and [18]F-FCH between the HER-negative/positive phenotypes, with a higher SUV for the HER2 −ve lesions (Table 5). The HER2 −ve phenotypes showed a higher semiquantitative value than the HER2 +ve phenotypes, which predicts the degree of intracellular metabolic reprogramming of an aggressive tumor.

Receiver operating curve to dichotomize the SUV_max_ for [18]F-FCH PET-CT in aggressive breast lesions (HER −ve group)

**Figure 6.**
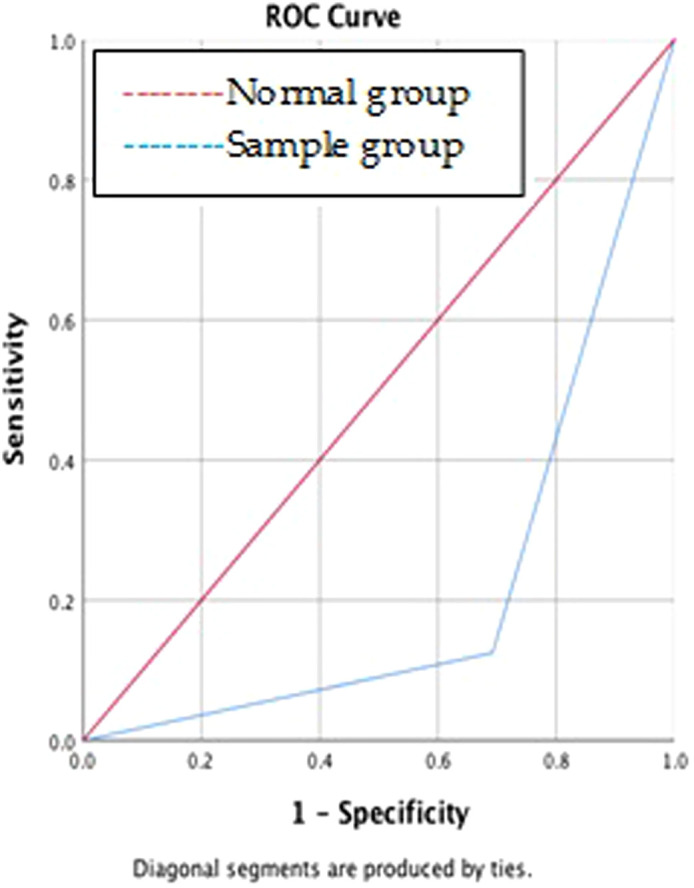
Receiver operating curve.

A cutoff value of 0.75 was deduced from the SUV_max_ among the [18]F-FCH breast cancer phenotype based on the best chances of sensitivity and specificity to predict the single ER/PG HER −ve group (Figure 6).

### Prediction of [18]F-Fluorocholine Positron Emission Tomography–Computed Tomography on the Quality of Life of Patients with Breast cancer

On follow-up of subjects at 6 months, there was a significant association between the dichotomized SUV_max_ and QOL domains (Table 6).

Table 6 shows the prediction of QOL of the PF by 18[F]-FCH at 6 months.

**Figure 7.**
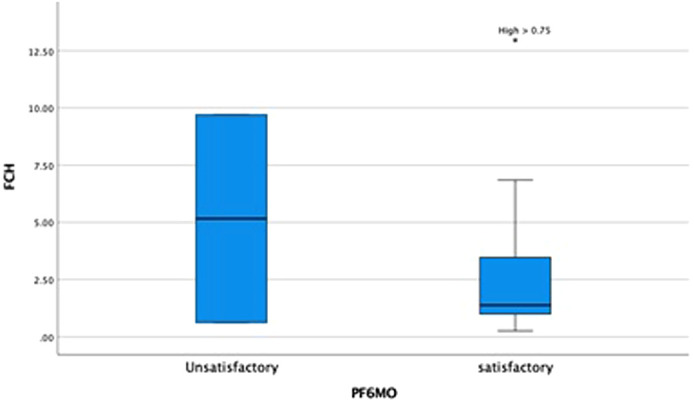
Plot graph showing the quality of life of physical function (PF) (satisfactory vs. unsatisfactory score and its association with maximum standardized uptake value [18]F-fluorocholine. Median; PF: 5.16 (SUV).

**Figure 8.**
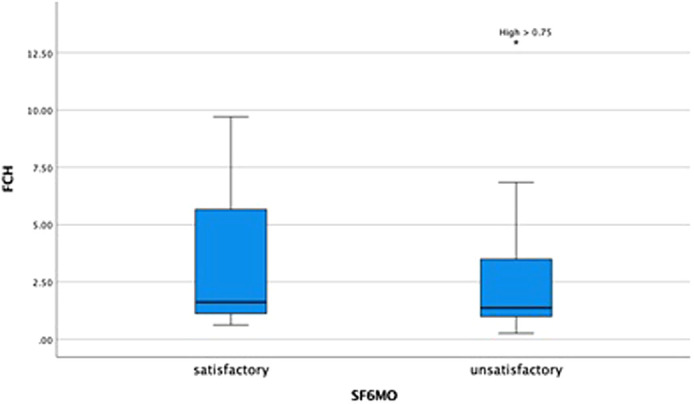
Plot graph showing the quality of life of social function (satisfactory vs. unsatisfactory score and its association with maximum standardized uptake value [18]F-fluorocholine. Median; physical function: 1.48 (SUV).

Physical function and social function were significantly disparity with dichotomized on [18]F-FCH SUV, with QOL scores of 8.067 and 3.267 (*P* < .05) (Table 6). There was no disparity between General Health Status and Role Function and FCH SUV. Likewise, there was no significant disparity between predicted SUV and QOL scores at 12 and 24 months (Table 7) or between [18]F-FDG PET and the QOL scores (Figure 7-8)

Table 7 shows the prediction of the QOL of GHS on the [18]F-FCH at 6, 12, and 24 months.

There was no significant disparity of the QOL scores with the [18]F-FCH SUV_max_ at 6, 12, or 24 months or between [18]F-FDG PET and the QOL scores.

### Quality of Life and Therapy

Eleven subjects had chemotherapy and targeted treatment, and 4 subjects were treated with surgery and radiation treatment. There was no significant association between the treatment types and the quality of life during follow-up across the time frames.

Table 8: Prediction of QOL on the treatment types at 6-month follow-up.

## Discussion

Breast cancer is a complex disease that requires a multimodality approach for diagnosis and treatment prediction. A dual PET tracer approach and multimodality imaging integrating PET-CT and magnetic resonance imaging (MRI) are promising diagnostic imaging techniques that would facilitate accurate patient management and indicate the need for surgery and toxic therapy. This study found high sensitivity and specificity of [18]F-FCH PET-CT in primary breast lesions of 40% and 68.8%, compared to [18]F-FDG PET-CT with 33.3% and 66.7% sensitivity and specificity, respectively. Some subcentimeter lymph node lesions can be attributed to small-volume sampling. This result is substantiated by another report stating that small-volume lesions were deemed a false adverse finding on PET.^18-19^ These findings align with the notion that [18]F-FCH could be a useful surrogate marker in staging, predicting tumor aggressiveness, and treatment outcomes of different tumor lineages, including breast cancer.^19-22^

[18]F-FCH may be a helpful marker in detecting distant metastasis, with significantly higher choline metabolic uptake (mean SUV_max_; 2.27 ± 3.19 g/dL) than glucose metabolism (mean SUV_max_: 1.74 ± 2.32 g/dL). Physiologically, the modulation and alteration of the enzymes that control the anabolic and catabolic pathways cause increased choline levels. Choline uptake increases in tumor tissue to keep up with the synthesis of phospholipids in cellular membranes. The raised and higher levels of choline-containing compounds are associated with cellular proliferation and altered glucose metabolism.^20-22^ Therefore, it is essential to avert a false negative malignant breast cancer that which harbors weak glutathione-1 expression as underpinned by the [18]F-FDG glucose signaling.

Single ER/PG HER2 −ve breast lesions have a poor prognosis and have been reported to mutate into aggressive lesions [18]. This study has documented an essential role of [18]F FCH in underpinning the cellular reprogramming of HER2 −ve phenotypes. We found that the mean SUV_max_ of FCH-PET was significantly higher in HER2 −ve than HER2 +ve lesions, as it conveys an aggressive cellular landscape. Notwithstanding this, the dominant and single ER/PG HER2 −ve distinct type features a potential molecular imaging predictor, i.e., choline metabolism of the cell membrane shown by PET-CT. On [18]F-FDG-PET signaling, Bae Sy et al (2015) et al found that single ER/PG in the HER2-negative breast cancer in 571 subjects was associated with poorer survival than single ER/PG HER2 +ve positive for which result is comparable to the Triple Negative Breast cancer.^23^ In our study, the SUV_max _of [18]F-FDG did not allow any significant differentiation between the 2 HER phenotypes. It could imply that the subcentimeter lesions are of greater intrinsic intensity on [18]F-FCH than [18]F-FDG imaging.

We also found that the cutoff value of SUV_max_ (g/dL) for [18]F FCH PET-CT could be used to predict the overexpression of the proliferative tumor lesion in the single ER/PG HER2 -ve phenotype. The SUV_max_ (g/dL) cutoff of 0.75 enabled stratification of the more aggressive breast lesions among the HER2 −ve group. In this regard, HER2 −ve lesions are less likely to respond favorably to hormonal therapy when this threshold exceeds 0.75 g/dL. Other groups have reported a cutoff value of 1.5 for positive [18]F-FDG-PET.^21,24,25^

The 21 subjects were followed up at 6, 12, and 24 months. The prediction of [18]F-FDG and [18]F-FCH on the quality of life scores were tested among the surviving subjects. In this cohort, a higher [18]F-FCH SUV_max_ predicted a poorer quality of life when the SUV_max_ value recidivated 0.75, particularly for PF)and SF at 6 months. There was no evidence that the SUV_max_ of [18]F-FDG was a significant predictor of the QOL (16) variables. Another study showed that a lower SUV_max_ of [18]F-FDG predicted prolonged survival and a higher quality of life among breast cancer patients.^26^

Our results show that [18]F FCH in PET-CT shows promise as a potential specific marker for detecting overexpression of the choline receptor metabolism, which underpins breast cancer aggressiveness. In other words, it could be used as a complementary marker to [18]F-FDG in localizing and predicting potentially poor FDG-avidity among the subcentimeter breast tumor lesions. The added value of [18]F-FCH in this regard is vital, given that the established use of [18]F FDG is deemed to be limited in small-volume lesions and tumor lineages with poor glutathione gene transporter expression [18]. In addition, [18]F FCH PET could be used to predict the quality of life among survivors.

The limitations of this study include the small sample size. However, the preliminary role of [18]F-FCH identified in this pilot cohort should encourage more extensive studies. Furthermore, the role of diffusion-weighted MRI would complement the dual tracer finding of [18]F-FDG–[18]F-FCH as the former may deduce the solid state of a lesion, which is commonly related to its high mitotic activities.^27^ The [18]F-FCH data’s role was based on a single time point as the study plan was kept close to the 18F FDG PET as a reference standard. There were time limitations for both studies at this juncture. In addition, the delayed image assessment analysis for the SUV_max_ has been reported on its usefulness for the 18F FDG in infection studies to distinguish infection from cancer. Nevertheless, there was no data on the delayed image for the 18F FCH, for which future studies should factor in this protocol to seek more evidence on the endpoints.

The utility of [18]F-FCH in PET-CT should be explored in the breast cancer cellular landscape given its heterogeneous cellular landscape, and the optimum utilization of the dual tracer approach may offer various clinical benefits, which will be necessary for devising personalized treatment in breast cancer patients. This study documented a preliminary outcome utilizing [18]F-FCH PET-CT as an essential marker to predict tumor aggressiveness in breast cancer.

## Figures and Tables

**Table 1.** Patient Demographics and Clinical Characteristics

Patient Characteristics	Count/SUV_max_ g/dL/Diameter (cm)	Percentage (%)
Age group (years)		
31-40	3	14.3
41-50	6	28.6
51-60	6	28.6
>61	6	28.6
BMI		
18.5-24.9	9	42.9
25.0-29.9	6	28.6
>30	6	28.6
Staging		
Stage I and II	8	38.1
Stage III and IV	13	61.9
Histology		
Invasive ductal carcinoma	18	85.7
Fibroadenoma	3	14.2
Overall mean SUV_max_; [18]F-FCH		
Single ER/PG (HER 2 −ve)	1.99 ± 1.52	
Single ER/PG (HER2 +ve); [18]F-FDG	0.20 ± 0.22	
Single ER/PG (HER 2 −ve)	2.95 ± 0.81	
Single ER/PG (HER2 +ve	2.79 ± 0.85	
Phenotype		
Single ER/PG (HER −ve)	10	52.4
Single ER/PG (HER2 +ve)	11	47.6

BMI, body mass index; HER2, human epidermal growth factor receptor 2; SUV, standardized uptake value.

**Table 2.** Sensitivity and Specificity of [18]F-Fluorocholine Positron Emission Tomography–Computed Tomography (PET-CT) Compared to [18]F-Fluorodeoxyglucose PET-CT in Breast, Lymph Node, and Metastasis

Imaging	Local		Lymph Node		Metastasis	
	Sensitivity	Specificity	Sensitivity	Specificity	Sensitivity	Specificity
[18]F-FDG	33.3%	66.7%	66.7%	83.3%	42.9%	92.9%
[18]F-FCH	40%	68.8%	44.5%	70%	27.3%	90%

Significant *P* < .05.

FCH, fluorocholine; FDG, fluorodeoxyglucose.

**Table 3.** [18]F-Fluorocholine and [18]F-Fluorodeoxyglucose Mean and Standard Deviation for Patients with Breast Lesions, Mixed-Side Lymph Node Lesions, and Metastasis

SUV_max_ mean	Local Right Breast (g/dL)	*P*	Local Left Breast (g/dL)	*P*	Mixed-Side Lymph Node (g/dL)	*P*	Metastasis (g/dL)	*P*
[18]F FDG	1.92 ± 0.97	.15	1.78 ± 0.99	.26	1.53 ± 1.7	0.32	1.74 ± 2.32	.004* (p less than 0.05)
[18]F FCH	1.34 ± 1.01		0.97 ± 0.81		1.28 ± 1.9		2.27 ± 3.19	

Significant *P* < .05.

FCH, fluorocholine; FDG, fluorodeoxyglucose; SUV, standardized uptake value.

**Table 4.** Association of the Overall Mean SUV_max_ of [18]F-Fluorocholine (g/dL) and [18]F-Fluorodeoxyglucose with Single ER/PG Human Epidermal Growth Factor Receptor 2 Status

**SUV_max_**	Single ER/PG HER2 −ve	Single ER/PG HER2 +ve	*P*
[18]F-FCH	1.99 ± 1.52	0.20 ± 0.22	<.05
[18]F-FDG	2.95 ± 0.81	2.79 ± 0.85	>.05

Significant *P* < .05.

FCH, Fluorocholine; FDG, Fluorodeoxyglucose; HER2, human epidermal growth factor receptor 2; SUV, standardized uptake value.

**Table 5.** Association of the Overall Standardized Uptake Value Mean of Single ER/PG Human Epidermal Growth Factor Receptors of R [18]F-Fluorodeoxyglucose and [18]F-Fluorocholine

SUV mean (g/dL)	HER2 −ve	HER2 +ve	*P*
[18]F-FDG	8.13 ±1.27	3.53 ± 3.51	<.01
[18]F-FCH	2.79 ± 0.85	0.22 ± 0.53	<.01

Significant *P*-value < .05.

FCH, fluorocholine; FDG, fluorodeoxyglucose; HER2, human epidermal growth factor receptor 2; SUV, standardized uptake value.

**Table 6.** Association of QOL (Quality of Life) of Physical Function and the 18[F]-Fluorocholine at 6 Months

****PF **Scores (High vs. Low)**	**GHS**	**PF**	**RF**	**SF**
SUV_max_ (high vs. low)	1.667	8.067	3.267	5.4
Asymp. Sig. (*P*)	.197	*.005	.071	*.02 (p is less than 0.05)

Score 1-5: poor-good. Significant when *P* < .05.

GHS, global health status; PF, physical function; RF, role function; SF, social function; SUV, standardized uptake value.

**Table 7.** Association Quality of Life of Global Health Status on the 18[F]-Fluorocholine at 24 Months

**GHS ** **Scores (High vs. Low)**	**GHS**	**PF**	**RF**	**SF**
SUV_max_ (high vs. low)	0.67	1.0	0.67	1.0
Asymp. Sig. (*P*)	.796	1.0	.796	1.0

Score 0: unsatisfactory; score 1: satisfactory. Significant when *P* < .05.

GHS, global health status; PF, physical function; RF, role function; SF, social function; SUV, standardized uptake value.

**Table 8.** Association Between Treatment Types and the Quality of Life at 6-Month Follow-Up

**GHS ** **Scores (High vs. Low)**	**GHS**	**PF**	**RF**	**SF**
SUV_max_ (high vs. low)	0.690	0.439	0.932	0.778
Asymp. Sig. (*P*)	.753	.661	.949	.851

GHS, global health status; PF, physical function; RF, role function; SF, social function; SUV, standardized uptake value; Score 0: unsatisfactory; 1: satisfactory.

Significant when *P* < .05.
